# Overexpression of *Brucella* putative glycosyltransferase WbkA in *B. abortus* RB51 leads to production of exopolysaccharide

**DOI:** 10.3389/fcimb.2015.00054

**Published:** 2015-06-24

**Authors:** Neha Dabral, Neeta Jain-Gupta, Mohamed N. Seleem, Nammalwar Sriranganathan, Ramesh Vemulapalli

**Affiliations:** ^1^Department of Comparative Pathobiology, College of Veterinary Medicine, Purdue UniversityWest Lafayette, IN, USA; ^2^Department of Biomedical Sciences and Pathobiology, Virginia-Maryland College of Veterinary Medicine, Virginia TechBlacksburg, VA, USA

**Keywords:** *B. abortus* RB51, glycosyltransferase, overexpression, exopolysaccharide, clumping

## Abstract

*Brucella* spp. are Gram-negative, facultative intracellular bacteria that cause brucellosis in mammals. *Brucella* strains containing the O-polysaccharide in their cell wall structure exhibit a smooth phenotype whereas the strains devoid of the polysaccharide show rough phenotype. *B. abortus* strain RB51 is a stable rough attenuated mutant which is used as a licensed live vaccine for bovine brucellosis. Previous studies have shown that the *wboA* gene, which encodes a glycosyltransferase required for the synthesis of O-polysaccharide, is disrupted in *B. abortus* RB51 by an *IS711* element. Although complementation of strain RB51 with a functional *wboA* gene results in O-polysaccharide synthesis in the cytoplasm, it does not result in smooth phenotype. The aim of this study was to determine if overexpression of *Brucella* WbkA or WbkE, two additional putative glycosyltransferases essential for O-polysaccharide synthesis, in strain RB51 would result in the O-polysaccharide synthesis and smooth phenotype. Our results demonstrate that overexpression of *wbkA* or *wbkE* gene in RB51 does not result in O-polysaccharide expression as shown by Western blotting with specific antibodies. However, *wbkA*, but not *wbkE*, overexpression leads to the development of a clumping phenotype and the production of exopolysaccharide(s) containing mannose, galactose, N-acetylglucosamine, and N-acetylgalactosamine. Moreover, we found that the clumping recombinant strain displays increased adhesion to polystyrene plates. The recombinant strain was similar to strain RB51 in its attenuation characteristic and in its ability to induce protective immunity against virulent *B. abortus* challenge in mice.

## Introduction

Members of the genus *Brucella* are Gram-negative, facultative intracellular coccobacilli that can cause chronic infections in several mammals, including humans. Based on the structure of the lipopolysaccharide (LPS) molecule and the colony morphology, *Brucella* spp. can be separated into smooth and rough phenotypes. Smooth colony morphology of *Brucella* strains is determined by the presence of LPS containing the O-polysaccharide (O-PS) in their cell wall structure. *Brucella* spp. with LPS that is devoid of the O-PS display rough colony morphology. Based on the reactive specificities of antibodies, the O-PS of *Brucella* smooth LPS is defined to contain A (for Abortus), M (for Melitensis), and C (for Common) epitopes (Diaz et al., 1968; Hurvell and Lindberg, 1973). *Brucella* O-PS is a linear homopolymer of 4,6-dideoxy-4-formamido-α-D-mannopyranosyl (perosamine) subunits connected in α-1,2 linkage in A-dominant smooth *Brucella* strains, with every fifth residue connected in α-1,3 linkage in M-dominant smooth *Brucella* strains (Caroff et al., 1984; Bundle et al., 1987). The O-PS is an immunodominant antigen, and infected animals usually develop robust antibodies to this antigen. Detection of anti-O-PS antibodies in the body fluids is the basis for several brucellosis diagnostic assays. At least in some animal species, anti-O-PS antibodies play a role in conferring enhanced protection against infections by *B. abortus, B. suis*, and *B. melitensis* (Araya and Winter, 1990; Ficht et al., 2009; Vitry et al., 2014). The O-PS also acts as a virulence factor by protecting the bacteria against complement-mediated lysis and the intracellular bactericidal milieu of phagocytic cells (Allen et al., 1998). Consequently, the smooth *Brucella* strains are generally more virulent than their rough counterparts, which are typically attenuated (Allen et al., 1998; McQuiston et al., 1999). *B. abortus* RB51, a laboratory derived stable rough attenuated strain, is used as a licensed live vaccine in the control of bovine brucellosis in the US and several other countries. Strain RB51 does not produce detectable levels of O-PS, and animals vaccinated with this strain do not develop anti-O-PS antibodies (Schurig et al., 1991). However, presence of low levels of M-like O-PS was detected in this strain (Cloeckaert et al., 2002).

The complete biosynthetic pathway of *Brucella* smooth LPS is yet to be determined. However, several genes are known to be essential for the biosynthesis of the O-PS (Godfroid et al., 2000; Gonzales et al., 2008; Zygmunt et al., 2009). These genes are located in two loci, *wbo* and *wbk*, on the *Brucella* chromosome (Zygmunt et al., 2009). Genes encoding four putative glycosyltransferases, *wboA, wboB, wbkA*, and *wbkE*, were identified to be involved in the polymerization of perosamine subunits leading to O-PS production (Godfroid et al., 2000; Gonzales et al., 2008; Zygmunt et al., 2009). The precise role of these four enzymes in O-PS synthesis is not yet established. In *B. abortus* RB51, the *wboA* gene is disrupted by an IS*711* element (Vemulapalli et al., 1999). Sequence analysis of the *wbo* and *wbk* loci of strain RB51 did not reveal any other gene-disrupting mutations (Adone et al., 2011). Complementation of strain RB51 with a functional *wboA* gene (RB51WboA) did not restore smooth phenotype, but resulted in the production of low levels of O-PS which remained in the cytoplasm (Vemulapalli et al., 2000). In mouse models, strain RB51WboA vaccination induced low titers of anti-O-PS antibodies and conferred superior protection against virulent *B. abortus* and *B. melitensis* challenge (Vemulapalli et al., 2000, 2004).

The initial objective of this study was to determine if overexpression of *wbkA* or *wbkE* in strain RB51 would overcome the *wboA* deficiency and lead to production of detectable levels of O-PS. We cloned each gene in a multi-copy plasmid under a strong synthetic promoter and used the resulting plasmids to transform strain RB51. Although we did not detect any O-PS production by the recombinant strains, unexpectedly, the overexpression of *wbkA*, but not *wbkE*, in strain RB51 led to the development of hypermucoid colonies and the production of exopolysaccharide(s) (EPS) containing mannose, galactose, N-acetylglucosamine, and N-acetylgalactosamine. The EPS producing strain was similar to strain RB51 in its attenuation and vaccine efficacy characteristics.

## Materials and methods

### Bacterial strains

*B. abortus* strains RB51 and 2308 were from our culture collection. *B. neotomae and Pseudomonas* were obtained from American Type Culture Collection. Generation of strain RB51WboA was described previously (Vemulapalli et al., 2000). *Escherichia coli* strain DH5α (Invitrogen, Carlsbad, CA) was used for the preparation of the necessary plasmid constructs. All bacteria were grown in tryptic soy broth (TSB) or on tryptic soy agar (TSA) at 37°C. Ampicillin at 100 μg/ml was used for growing bacteria harboring plasmids. All experiments with virulent *Brucella* were performed in a BSL-3 facility approved for the select agents work.

### Generation of recombinant strains RB51WbkA and RB51WbkE

The *wbkA* and *wbkE* genes were amplified by PCR using custom-designed primer-pairs and the genomic DNA of *B. abortus* 2308 as template. For the *wbkA* gene, the forward primer (5′-TT*TTCCATGG*CTCCCTACGAATACATTTGCA-3′) and the reverse primer (5′-TTTT*TCTAGA*TTAATAGGTCATGAGCTTAGATTC-3′) contained *Nco* I and *Xba* I restriction sites, respectively, at the 5′ ends. Similarly, for the *wbkE* gene, the forward primer (5′-*AAGCTT*ATGCCGCATCTGTATTGGAGA-3′) and the reverse primer (5′-*GGATCC*TCACTGCATCAGCGACGTATA-3′) contained *Hind* III and *Bam* HI restriction sites, respectively, at the 5′ ends. The amplified fragments were first cloned in pGEM-T Easy plasmid (Promega, Madison, WI) and sequenced to confirm the integrity of their nucleotide sequences. The inserts were subsequently excised from the pGEM-T plasmids using the restriction enzymes specific to the respective restriction sites engineered into the primers and cloned in the same sites of pBB4Trc plasmid (Kovach et al., 1995). The resulting plasmids, pBB4TrcWbkA and pBB4TrcWbkE, were electroporated into strain RB51 as per previously described procedure (McQuiston et al., 1995) to generate strains RB51WbkA and RB51WbkE, respectively.

### RNA isolation and reverse transcriptase (RT)-PCR

Cultures of RB51, RB51WbkA, and RB51WbkE were grown in TSA at 37°C. Ampicillin at 100 μg/ml was used for growing bacteria harboring plasmids. Total RNA was isolated from the bacteria using the RNAeasy total isolation kit (Qiagen Inc., Valencia, CA) according to the manufacturer's protocol. Contaminating DNA was removed using Turbo DNA-free™ kit (Life Technologies, Grand Island, NY) following manufacturer's instructions. RNA purity and concentration were evaluated using electrophoresis and the Nanodrop® ND-1000 (Nanodrop, Wilmington, DE).

The reverse transcription assay was used to quantify the expression levels of *wbkA* and *wbkE* mRNA in different strains. 100 ng of total RNA was reverse transcribed into cDNA and amplified in a single step using Superscipt III Platinum SYBR green one-step qRT-PCR kit (Life Technologies, Grand Island, NY). Gene specific primers were used for the RT-PCR assay. For *wbkA* gene, the forward primer (5′-TT*TTCCATGG*CTCCCTACGAATACATTTGCA-3′) and the reverse primer (5′-CTCGAAAGTCGAATCCTTCTGAAGAGGATAAC-3′) were used. Similarly, for the *wbkE* gene, the forward primer (5′-*AAGCTT*ATGCCGCATCTGTATTGGAGA-3′) and the reverse primer (5′-TCGGGTATTTTCTTCCGGCTTTGCAC-3′) were used. To normalize the *wbkA and wbkE* gene expression, we used the gene for the translation initiation factor IF-1 of *B. abortus* (Hernandez-Castro et al., 2003). For IF-1, the forward primer (5′-ATGGCGAAAGAAGTCCT-3′) and the reverse primer (5′-ACTAGAACCTTGTCACCGGC-3′) were used. The RT-PCR assay was performed in triplicates for each sample using Stratagene MX3000P thermocycler (Stratagene, La Jolla, CA). The samples were incubated at 50°C for 15 min for cDNA synthesis. After another incubation step at 95°C for 5 min, the samples were subjected to 40 cycles (30 s at 95°C, 1 min at 55°C, 30 s at 72°C). The fold change was calculated using the comparative threshold method (Livak and Schmittgen, 2001).

### Sample preparation for electron microscopy

Freshly grown cultures of strains RB51 and RB51WbkA were mixed with an equal volume of stock buffer (0.1 M cacodylate buffer, pH 6.8) and incubated at room temperature for 10 min. The bacterial cells were then pelleted by centrifugation and resuspended in primary fixative solution (2.5% glutaraldehyde and 2% paraformaldehyde in 0.1 M stock buffer, pH 6.8). After 1 h of incubation, the cells were washed two times by centrifugation with the stock buffer, followed by a final washing with water. Secondary fixation of the bacteria was performed for 1 h in a solution containing 1% osmium oxide and 1.5% potassium ferricyanide.

#### Scanning electron microscopy

After the secondary fixation, the bacterial cells were washed two times with water and filtered using a nucleopore membrane (25 mm diameter, 0.2 mm pore size, Corning corp., 45 Nagog Park, Acton, MA). The bacterial cells were dehydrated using increasing concentrations of ethanol and the samples were mounted using a double-side carbon tape and sputter coated with platinum (Pt) for 60 s prior to imaging. Images were obtained using FEI NOVA nanoSEM (FEI Company, Portland OR) with 5 kV accelerating voltage.

#### Transmission electron microscopy

After the secondary fixation, the bacterial cells were washed two times with water and pelleted by centrifugation. Melted (45°C) agarose (1.5% w/v) was added to the tube and the bacterial cells were gently dispersed while keeping the tube in warm water. The dispersed bacterial cells and agarose mixture was cooled and extracted from the tube using 10% ethanol. Samples were then sliced and dehydrated using increasing concentrations of ethanol. Propylene oxide (PO) was used for a final rinse. Infiltration was carried out with 1/3 Spurr's resin (3 parts PO: 1 part resin) overnight, followed by further infiltration with 1/1 Spurr's resin (1 part PO: 1 part resin), 3/1 Spurr's resin (1 part PO: 3 parts resin) overnight, and finally with 100% Spurr's resin for 6 h in a rotator. The cells were then embedded in a fresh Spurr's resin and polymerized for 2 days at 60°C. Samples were viewed under the FEI/Philips CM-10 transmission electron microscope (FEI Company, Hillsboro, OR) using an accelerating voltage of 80 kV.

### SDS-PAGE and western blotting

To detect O-polysaccharide expression and compare the protein profiles of RB51 and its recombinant strains, SDS-PAGE and Western blot analyses were performed as previously described (Vemulapalli et al., 1998). As controls, antigen extracts of the strains RB51, RB51WboA, and *B. neotomae* were used. Briefly, cultures of RB51, RB51WbkE, RB51WboA, and *B. neotomae* were grown in TSB at 37°C. Strain RB51WbkA was grown in TSA at 37°C. Ampicillin at 100 μg/ml was used for growing bacteria harboring plasmids. One ml of the culture at OD_600_ = 2.0 was centrifuged at 14,000 rpm for 2 min and the pellet was resuspended in 50 μl of 2X Laemmli buffer. Strain RB51WbkA was scrapped from the plate and suspended in distilled water and processed as above. The resuspended pellets were heated in boiling water for 2–5 min and centrifuged at 14,000 rpm for 10 min. The clear supernatants were loaded into wells of a 12.5% denaturing polyacrylamide gel and the antigens were separated by electrophoresis and stained with Coomassie Brilliant blue. For Western blotting, the separated antigens were transferred onto a nitrocellulose membrane which was subsequently blocked with 5% skim milk and reacted with an appropriately diluted rat monoclonal antibody specific to *Brucella* O-PS (Schurig et al., 1991). The bound primary antibody was detected by reacting with horseradish peroxidase labeled-secondary antibody (KPL, Gaithersburg, MD), and developing the enzyme reaction using a colorimetric substrate (TMB substrate, KPL, Gaithersburg, MD).

### Exopolysaccharide (EPS) staining

Recombinant strain RB51WbkA and the strain RB51 were grown for 24 h at 37°C in TSB with ampicillin and TSB alone, respectively. The bacteria were fixed with 4% paraformaldehyde (PFA) for 20 min and used for staining.

#### Calcofluor white staining

For detection of polysaccharides, the fixed cells were washed three times with phosphate-buffered saline (PBS) (pH 8.5) and resuspended in 100 μl of the same buffer. 10 μl of the cell preparation was placed on a slide and one drop of calcofluor white stain (Fluorescent whitener 28, Sigma) was added to the cells. A coverslip was placed over the sample and the cells were visualized immediately using a Nikon A1R confocal laser scanning microscope with a 60X 1.4 NA oil immersion objective.

#### Lectin staining of EPS

Fluorescently labeled lectins (Vector laboratories Inc, Burlingame, California), conjugated with tetramethylrhodamine isothiocyanate (TRITC), with different sugar specificities (Table 1) were used to characterize the EPS composition. Fixed RB51 and RB51WbkA bacterial cells were stained with TRITC-labeled lectins (20 μg/ml in PBS). 4′,6-diamidino-2-phenylindole (DAPI) was used to counterstain the bacterial cells. After an incubation for 30 min in the dark at room temperature, the cells were washed three times with PBS, resuspended in 100 μl of the same buffer, and examined immediately using a Nikon A1R confocal laser scanning microscope with a 60X 1.4 NA oil immersion objective.

**Table 1:** **Lectin-binding specificities of EPS(s) produced by the recombinant strain RB51WbkA**.

**Lectin**	**Abbreviation**	**Primary sugar specificity**	**Binding with strain RB51**	**Binding with strain RB51WbkA**
*Griffonia simplicifolia* lectin I	GSL I	Galactose	±	+++
*Lens culinaris* lectin	LCA	Mannose	+	+++
*Phaseolus vulgaris* erythroagglutinin	PHA-E	Complex structures	−	−
*Phaseolus vulgaris* leucoagglutinin	PHA-L	Complex structures	−	−
*Pisum sativum* agglutinin	PSA	Mannose	+	+++
Wheat germ agglutinin, succinylated	Succinylated WGA	N-acetylglucosamine	±	+++
*Sophora japonica* agglutinin	SJA	N-acetylgalactosamine	±	+++

### Competitive inhibition assay

The sugars D-(+)-galactose, D-(+)-mannose, *N*-acetyl-D-galactosamine and *N*-acetyl-D-glucosamine (all from Sigma) were used to evaluate the carbohydrate binding specificity of the selected lectins (see Table 1). The sugars at a final concentration of 3 mg/ml or 100 mg/ml were mixed with solutions containing specific lectins at a concentration of 20 μg/ml. The mixtures were incubated for 30 min in dark at room temperature to allow the sugars to bind with specific lectins. Each sugar plus lectin mixture was then used for staining the bacterial cells as described above.

### Microtiter plate attachment assay

The attachment assay was performed as previously described (Djordjevic et al., 2002), with few changes. Briefly, strain RB51WbkA was freshly grown overnight in 10 ml of TSB with ampicillin at 37°C. As controls, strain RB51 and *Pseudomonas* were grown overnight in 10 ml of TSB at 37°C. Hundred micro liter of the overnight cultures was transferred to 10 ml of TSB, mixed thoroughly by vortexing, and 200 μl of each resuspended culture was transferred to eight wells in a 96-well polystyrene plate (USA Scientific, Ocala, FL). The plates were incubated at 37°C for 20 h. Then, the liquid medium was removed and the attached cells were washed with sterile PBS (pH 7.4). Plates were air dried for 45 min and each well was stained with 150 μl of 1% crystal violet solution (GRAM'S solution, Merck) in water for 45 min. The wells were then rinsed with water, air dried, and the bound stain was released by adding 200 μl of 95% ethanol. 100 μl from each well was transferred to a new microtiter plate and the intensity of the color was determined by reading the absorbance at 595 nm in a spectrophotometer (Molecular devices, Sunnyvale, CA).

### Survival of strain RB51WbkA in mice

Female BALB/c mice of 4 to 6 weeks of age were used. Groups of nine mice were immunized by intra-peritoneal (i.p.) inoculation with 2 × 10^8^ CFU-equivalent of strains RB51 or RB51WbkA. At days 1, 7, and 21 post immunization (p.i.), three mice from each group were euthanized by CO_2_ asphyxiation followed by cervical dislocation. The spleens were collected aseptically and the *Brucella* CFUs per spleen were determined as previously described (Schurig et al., 1991). Briefly, the spleens were homogenized in TSB and ten-fold serial dilutions of the homogenates were plated on TSA plates for RB51 and TSA plates containing ampicillin for RB51WbkA. The bacterial CFUs were enumerated.

### Mice immunization and serum collection

Four female BALB/c mice of 4 to 6 weeks of age were immunized by i.p. inoculation at day 0 with 2 × 10^8^ CFU-equivalent of RB51 or RB51WbkA. Mice inoculated with saline served as control. Blood was collected from the mice by puncturing the retro-orbital plexus under anesthesia at 3 and 6 weeks p.i. The serum was separated from the clotted blood and stored at −20°C until further use for the detection of antigen-specific antibodies by indirect enzyme-linked immunosorbent assay (ELISA).

### Indirect ELISA

Indirect ELISA was used to determine the levels of serum immunoglobulin G (IgG), as well as IgG1, IgG2a, IgG2b, and IgG3 isotypes with specificity to whole antigens of RB51 and RB51WbkA. Prior to coating the plates for ELISA, RB51, and RB51WbkA were heat-killed by incubating at 65°C for 1 h. The antigens were diluted in carbonate buffer, pH 9.6, to a final concentration of 1 × 10^8^ CFU-equivalent ml^−1^. The wells of polystyrene plates (Nunc-Immunoplate with maxisorp surface) were coated with the diluted antigens (100 μl/well). Following overnight incubation at 4°C, the plates were washed four times in wash buffer (TBS at pH 7.4, 0.05% Tween 20) and blocked with 5% skim milk in TBS. After 1 h of incubation at 37°C, mouse sera at 1 in 100 dilution in blocking buffer were added to the wells (50 μl/well). Each serum sample was tested in duplicate wells. Following incubation at room temperature for 4 h, the plates were washed four times in wash buffer and appropriately diluted horseradish peroxidase-labeled anti-mouse isotype specific conjugates (Southern Biotechnology Associates Inc, Birmingham, Alabama) were added to the wells (50 μl/well). After further 1 h incubation at room temperature, the plates were washed four times, and 100 μl of substrate solution (TMB Microwell peroxidase substrate; KPL, Gaithersburg, MD) was added to each well. After 20 min, the enzyme reaction was stopped by adding 100 μl of stop solution (0.185 M sulfuric acid) and the absorbance at 450 nm was recorded using microplate reader (Molecular devices, Sunnyvale, CA).

### Protection experiment

Groups of five female BALB/c mice of 4–6 weeks of age were vaccinated by i.p. inoculation with 2 × 10^9^ CFU-equivalent of RB51 or RB51WbkA. A group of mice inoculated with saline alone served as a control. Seven weeks p.i, each mouse was challenged by i.p. inoculation with 3 × 10^4^ CFU-equivalent of *B. abortus* 2308. Two weeks post-challenge, the mice were euthanized and the bacterial burden in their spleens was enumerated as previously described (Schurig et al., 1991).

### Statistical analyses

Absorbance values of ELISA were analyzed for differences among the groups by performing analysis of variance with *post-hoc* Bonferroni and Tukey for pair-wise comparison using SPSS version 21.0 (SPSS Inc., an IBM company, USA). For protection study, Student *t*-test modified for unequal variances between groups was performed to compare the log transformed bacterial loads in spleens of mice from each vaccinated group with the respective saline group. *P* < 0.01 were considered significant.

### Ethics statement

The protocols of the mice experiments performed in this study were approved by the Institutional Animal Care and Use Committees at Purdue University (Approval # 1112000488) and Virginia Tech (Approval # CVM-10-048). The animal studies were conducted in strict accordance with the recommendations in the Guide for the Care and Use of Laboratory Animals of the National Institutes of Health. Blood was collected from the retro-orbital plexus from mice under anesthesia. For anesthetizing mice, regulated concentration of anesthetic mixture (oxygen and isoflurane) was administered via a commercially available rodent anesthesia machine (Vetamac, Inc., Rossville, Indiana). Following blood collection, a drop of proparacaine hydrochloride ophthalmic solution (Bausch & Lomb, Tampa, Florida) was placed on the eye to reduce pain. Mice infected with virulent *B. abortus* 2308 do not develop clinical disease or exhibit any signs of suffering for the duration of the experiments conducted in this study. Therefore, no humane endpoints were utilized for the mice in this study.

## Results

### *B. abortus* RB51 overexpressing *wbkA* gene displays a clumping phenotype

**Figure 1:**
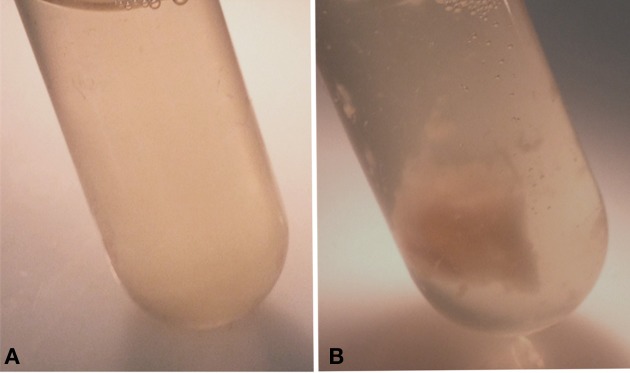
**Observation of the clumping phenotype of the recombinant strain** ***B. abortus*****RB51WbkA. (A)** *B. abortus* RB51; **(B)** Strain *B. abortus* RB51 overexpressing the *wbkA* gene.

The colonies of strain RB51WbkA exhibited an excessively mucoid phenotype on agar plates and when grown in liquid culture, the bacteria formed strings and clumps (Figure 1B). Strains RB51 (Figure 1A) and RB51WbkE (data not shown), on the other hand, displayed uniform dispersion in liquid culture. As a result of the strikingly distinctive phenotype of strain RB51WbkA, several colonies were analyzed by RB51-specific PCR (Vemulapalli et al., 1999) and all of them were confirmed to be derived from strain RB51 (data not shown).

**Figure 2:**
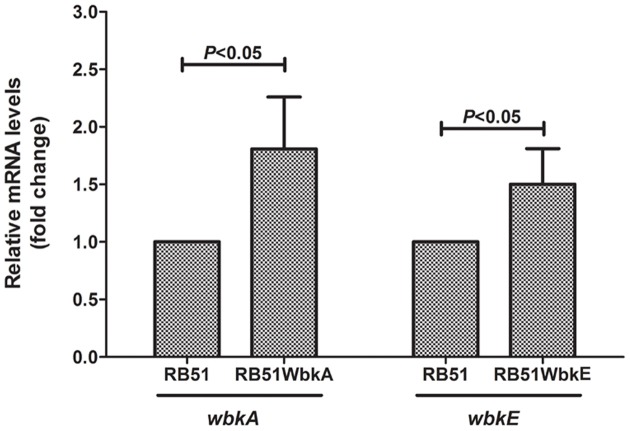
**RT-PCR detection of** ***wbkA*** **and** ***wbkE*** **mRNA in strains RB51, RB51WbkA, and RB51WbkE**. Graph shows the mean values of fold change for *wbkA* and *wbkE* transcript levels in the recombinant strains RB51WbkA and RB51WbkE, respectively, relative to those of RB51, which were converted to 1. All the values are relative to those of internal control gene IF-1. Results are shown as mean ± standard deviation (*n* = 3).

RT-PCR assay was performed to quantify the levels of *wbkA* and *wbkE* mRNA in different strains. An increase of ≈two-fold was observed in the expression level of *wbkA* in strain RB51WbkA relative to the RNA level observed in RB51 (Figure 2). Also, the expression level of *wbkE* was increased ≈1.5 fold in strain RB51WbkE when compared to the RNA expression level in strain RB51 (Figure 2). Due to the distinct mucoid phenotype of RB51WbkA, one colony was selected for further studies.

**Figure 3:**
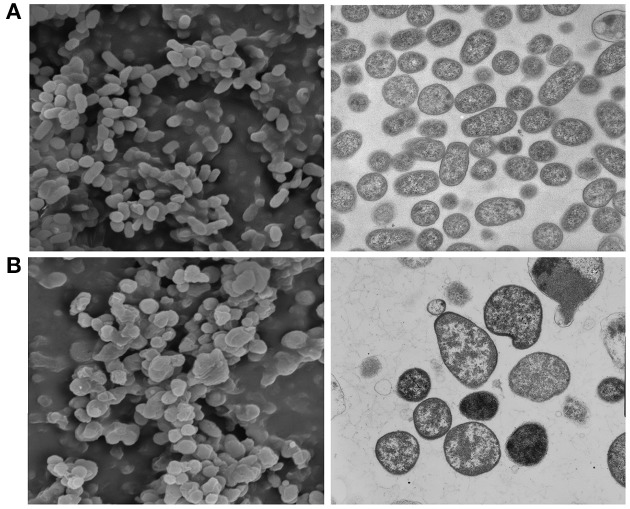
**The recombinant strain RB51WbkA displays a clumping phenotype and produces EPS(s)**. Scanning electron micrographs (left panels; X30,000) and transmission electron micrographs (right panels; X15,000) of the strains **(A)** *B. abortus* RB51 and **(B)** *B. abortus* RB51WbkA. Scanning electron micrograph show the clumping phenotype of the recombinant strain RB51WbkA (**B**, left panel).

Scanning and transmission electron microscopy were performed to examine the morphology of the bacteria. Strain RB51 culture contained well-defined coccobacilli, with relatively little or no visible extracellular material (Figure 3A). In contrast, the recombinant strain RB51WbkA displayed formation of aggregates containing bacterial cells and extraneous matrix material (Figure 3B, left panel) characteristic of EPS. The recombinant bacterial cells also displayed altered cell walls (Figure 3B).

### Overexpression of *wbkA* in the strain RB51 does not result in O-PS synthesis

**Figure 4:**
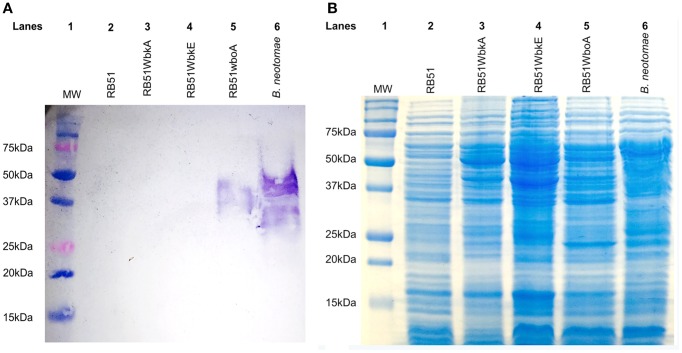
**Detection of O-PS expression and difference in the protein profiles of strains RB51, RB51WbkA, RB51WbkE, RB51WboA, and** ***B. neotomae***. Whole antigens of the strain RB51, RB51WbkA, RB51WbkE, RB51WboA, and *B. neotomae* were separated by 12.5% SDS-PAGE and analyzed by **(A)** Western blotting with O-PS specific monoclonal antibody, Bru-38; and by **(B)** Coomassie brilliant blue staining of polyacrylamide gel. Lane 1 in both panels contains molecular weight markers (MW) as indicated in kilodaltons (kDa).

The antigen extracts of strain RB51WbkA did not react with Bru-38, a *Brucella* O-PS-specific monoclonal antibody, indicating the absence of O-PS expression (Figure 4A, lane 3). As expected, strain RB51WboA and *B. neotomae* reacted with the antibody (Figure 4A, lanes 5 and 6, respectively), while no reaction was detected with strain RB51 (Figure 4A, lane 2). Strain RB51WbkE also did not react with the O-PS specific monoclonal antibody (Figure 4A, lane 4).

SDS-PAGE analysis did not reveal any apparent qualitative differences in the protein profiles between strains RB51 and RB51WbkA (Figure 4B).

### RB51WbkA bacterial clumps contain EPS

**Figure 5:**
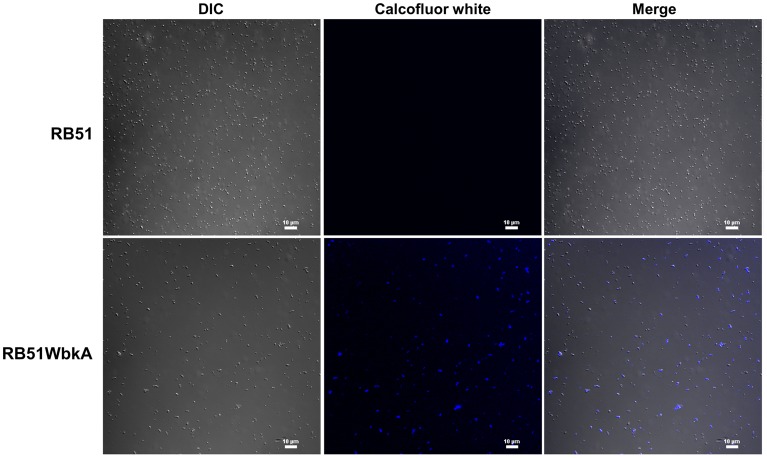
**Identification of the EPS(s) produced by the recombinant strain RB51WbkA**. Interactions between the calcofluor white stain and the aggregates formed by the strain RB51WbkA were visualized using confocal laser scanning microscopy. Selected differential interference contrast (DIC) images (left panel) and fluorescent images (middle panel) merged are shown (right panel). All images were acquired using the same settings and adjusted for display using the same brightness/contrast settings.

**Figure 6:**
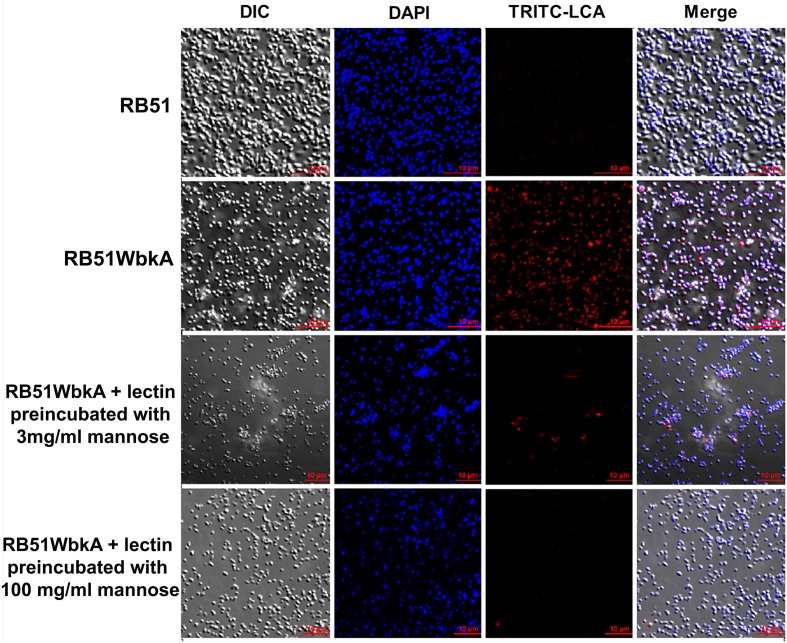
**Interactions between the fluorescently labeled LCA lectin and the strains RB51 and RB51WbkA are visualized using confocal laser scanning microscopy**. The bacterial cells are stained with TRITC-labeled LCA lectin (red) in the absence of the target carbohydrate, or preincubated with 3 mg/ml mannose or 100 mg/ml mannose, prior to staining. DAPI (blue) was used to stain the bacterial nuclei. Selected differential interference contrast (DIC) images (left panel) and fluorescent images (middle panels) merged are shown (right panel). All images were acquired using the same settings and adjusted for display using the same brightness/contrast settings.

**Figure 7:**
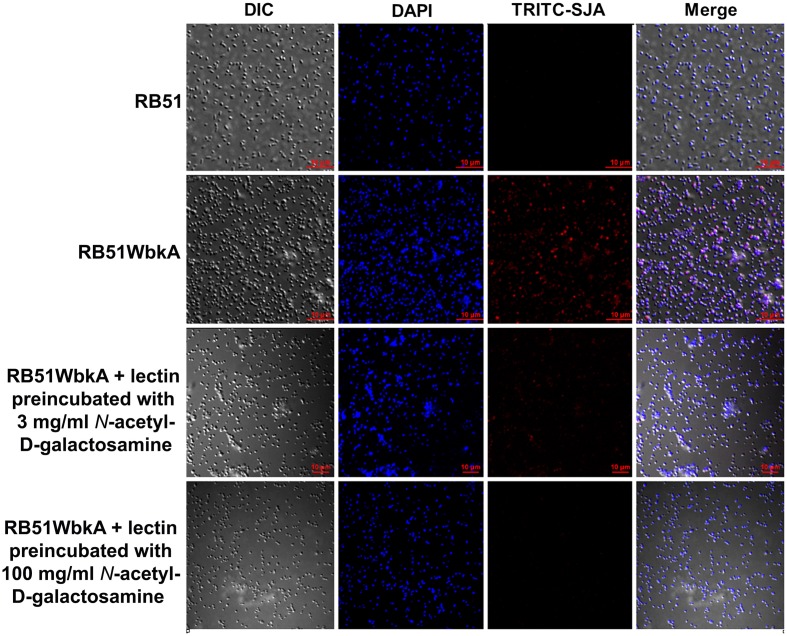
**Interactions between the fluorescently labeled SJA lectin and the strains RB51 and RB51WbkA are visualized using confocal laser scanning microscopy**. The bacterial cells are stained with TRITC-labeled SJA lectin (red) in the absence of the target carbohydrate, or preincubated with 3 mg/ml N-acetylgalactosamine or 100 mg/ml N-acetylgalactosamine, prior to staining. DAPI (blue) was used to stain the bacterial nuclei. Selected differential interference contrast (DIC) images (left panel) and fluorescent images (middle panels) merged are shown (right panel). All images were acquired using the same settings and adjusted for display using the same brightness/contrast settings.

Bacterial extracellular matrices are frequently composed of polysaccharides. Therefore, a general EPS dye, calcofluor white, was used to determine the presence of EPS in the aggregate-forming strain RB51WbkA. As shown in Figure 5, a bright fluorescence was exhibited by strain RB51WbkA, indicating the presence of an extracellular polysaccharide composed of (1-4)- and/or (1-3)-β-D-linked glucan residues. The dye failed to bind with strain RB51 (Figure 5). The bacterial cells of strain RB51WbkA also bound with mannose-specific-TRITC-labeled LCA (Figure 6), mannose-specific-TRITC-labeled PSA (data not shown), galactose-specific-TRITC-labeled GSL I (data not shown), N-acetylglucosamine-specific-TRITC-labeled succinylated WGA (data not shown) and N-acetylgalactosamine-specific-TRITC-labeled SJA (Figure 7). However, strain RB51WbkA did not demonstrate any apparent binding to the lectins PHA-E and PHA-L (data not shown), which have specificity for complex polysaccharide structures. The bacterial cells of strain RB51 exhibited a weak fluorescence with mannose-specific-TRITC-labeled LCA (Figure 6) and PSA lectins (data not shown) when compared to strain RB51WbkA. Also, strain RB51 demonstrated negligible binding with the other lectins tested, including TRITC-labeled SJA (Figure 7). The lectin-binding specificities of strains RB51 and RB51WbkA are shown in Table 1.

### EPS produced by RB51WbkA contains mannose, galactose, N-acetylglucosamine, and N-acetylgalactosamine

In order to verify the specific lectin-binding pattern of strain RB51WbkA, competitive inhibition assay was carried out using five selected lectins (TRITC conjugated LCA, PSA, WGA, SJA, and GSL I). Carbohydrate inhibition of the binding of specific TRITC-labeled lectins to the EPS components was accomplished by using their respective target primary sugars (Table 1). The inhibition of lectin binding was evaluated microscopically by comparing the binding characteristic as well as the fluorescence intensity of strain RB51WbkA in presence and absence of the target carbohydrate. The observed binding pattern of the TRITC-labeled LCA and SJA are shown (Figures 6, 7, respectively). Original binding pattern of the TRITC-labeled LCA and SJA to the EPS(s) of the strain RB51WbkA in the absence of target carbohydrate is shown in Figures 6, 7, respectively. Binding of the TRITC-labeled lectins to the EPS(s) was completely abrogated when the lectins were incubated with their target sugars at a concentration of 100 mg/ml (Figures 6, 7). At carbohydrate concentrations of 3 mg/ml, the binding of TRITC-labeled LCA (Figure 6), PSA (data not shown), WGA (data not shown), and GSL I (data not shown) to the bacterial strain RB51WbkA was greatly reduced when compared with the binding pattern in the absence of the target sugars (Figure 6). However, at 3 mg/ml, N-acetylgalactosamine only slightly inhibited the binding of TRITC-labeled SJA lectin to the EPS(s) produced by strain RB51WbkA (Figure 7).

### RB51WbkA displays increased adhesion property

**Figure 8:**
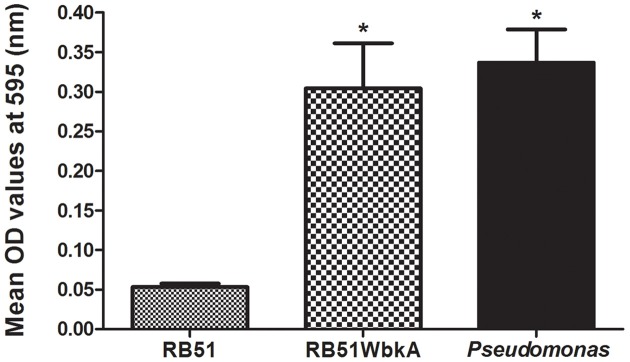
**The recombinant strain RB51WbkA displays increased adhesion property**. The surface attachment of the strain RB51, recombinant strain RB51WbkA, and *Pseudomonas* spp. is shown. Quantification of the microtiter plate adhesion is done by staining the adherent bacterial cells with crystal violet, subsequent solubilization of the crystal violet stain in ethanol and spectrophotometric determination of absorbance at 595 nm. Results are shown as mean ± standard deviation of absorbance of eight independent samples. ^*^Significantly different from the RB51 strain (*P* < 0.01).

EPS is a key component of biofilms in many bacteria and has been found to be essential for virulence; it plays a crucial role in adhesion and colonization, resistance to phagocytosis as well as immune evasion toward antibacterial peptides (Vyong et al., 2004; Flemming et al., 2007). We assessed the ability of strain RB51WbkA to adhere to a 96-well polystyrene plate when compared to strain RB51. *Pseudomonas* spp. was used as a positive control for this adherence assay. Strain RB51WbkA displayed significantly increased adherence to polystyrene wells when compared to strain RB51 (Figure 8).

### Bacterial persistence in mice spleens

**Figure 9:**
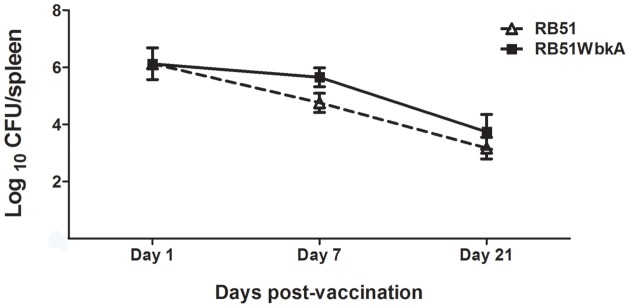
**Overexpression of** ***wbkA*** **in RB51 does not result in increased survival in mice**. Mice spleens were collected at days 1, 7, and 21 after vaccination with RB51 and recombinant RB51WbkA. The *Brucella* CFUs in their spleens were determined. Results are shown as mean ± standard deviation (*n* = 3) of the log CFU of *Brucella* recovered from spleens.

Bacterial persistence of strain RB51WbkA in mouse spleens was determined and compared with that of strain RB51. As shown in Figure 9, similar numbers of bacteria were present in the spleens of mice at days 1, 7, and 21 after inoculation with strains RB51WbkA and RB51. This result suggests that overexpression of *wbkA* did not affect the clearance of the recombinant RB51 strain in mice. Also, the mucoidal phenotype of the strain RB51WbkA was stable after *in vivo* passage.

### Induction of antigen-specific antibody responses

**Figure 10:**
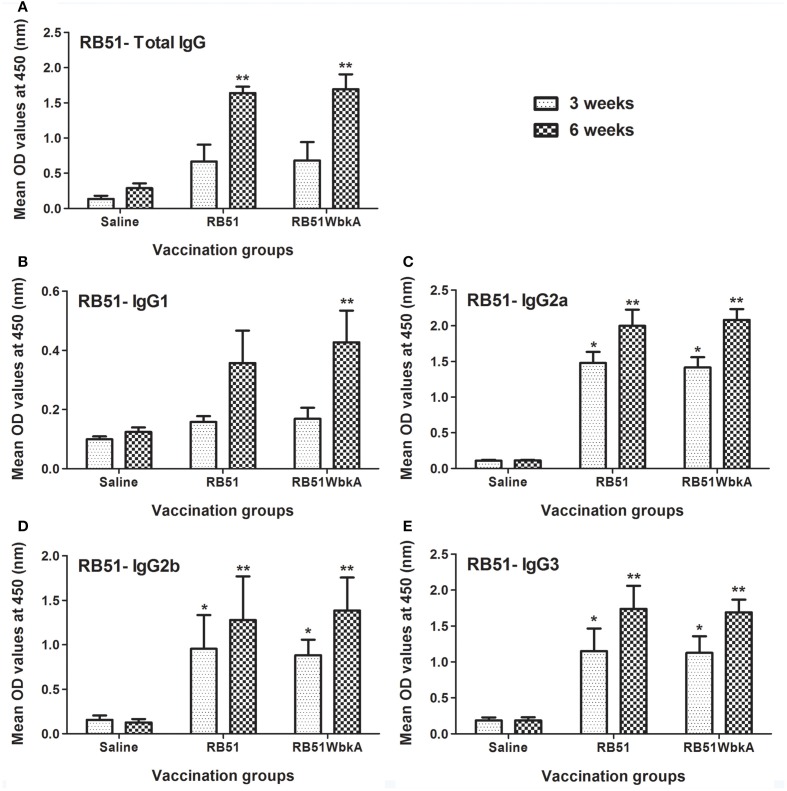
**Detection of RB51-specific (A) IgG, (B) IgG1, (C) IgG2a, (D) IgG2b, and (E) IgG3 antibodies in serum of mice vaccinated with live RB51, live recombinant RB51WbkA and saline-inoculated mice**. Serum samples were collected at 3 and 6 weeks after vaccination, were diluted 1 in 100 and assayed for the presence of total antigen-specific antibodies by indirect ELISA. Results are shown as mean ± standard deviation (*n* = 4) of absorbance of the color developed. ^*^Significantly different from the corresponding saline group at week 3 (*P* < 0.01). ^**^Significantly different from the corresponding saline group at week 6 (*P* < 0.01).

**Figure 11:**
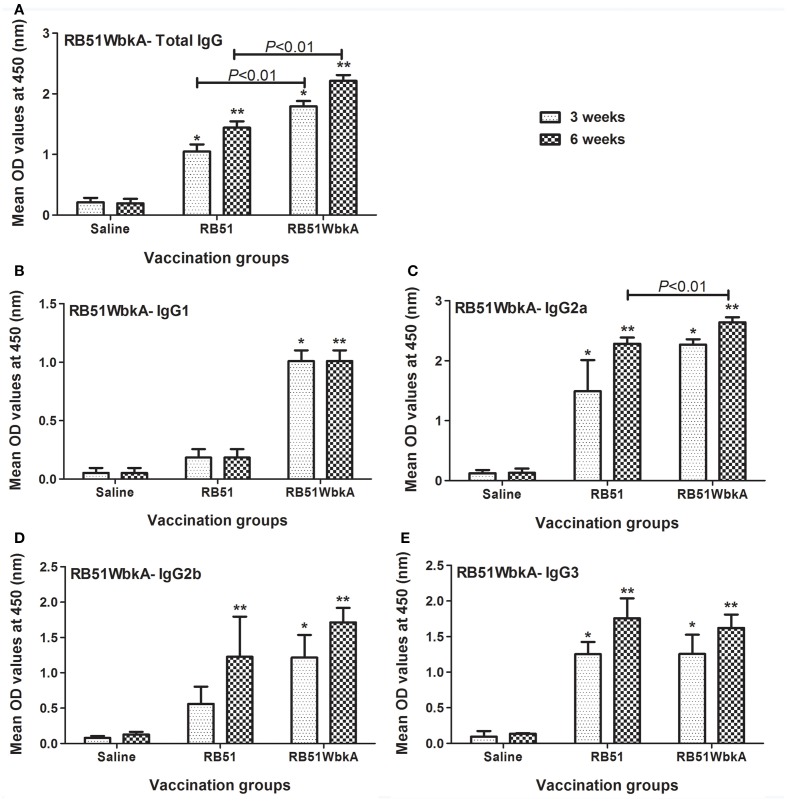
**Detection of RB51WbkA-specific (A) IgG, (B) IgG1, (C) IgG2a, (D) IgG2b, and (E) IgG3 antibodies in serum of mice vaccinated with live RB51, live recombinant RB51WbkA and saline-inoculated mice**. Serum samples were collected at 3 and 6 weeks after vaccination, were diluted 1 in 100 and assayed for the presence of total antigen-specific antibodies by indirect ELISA. Results are shown as mean ± standard deviation (*n* = 4) of absorbance of the color developed. ^*^Significantly different from the corresponding saline group at week 3 (*P* < 0.01). ^**^Significantly different from the corresponding saline group at week 6 (*P* < 0.01).

EPS constituents represent major surface antigens in many bacteria and have been found to be of great importance in the development of vaccines against various infectious diseases (Amorena et al., 1994; Pier et al., 1994; Flemming et al., 2007; Prenafeta et al., 2010). To determine the immunogenicity of strain RB51WbkA, serum samples collected from the groups of mice immunized with strains RB51 and RB51WbkA were analyzed in comparison with the sera obtained from the saline-inoculated group of mice (Figures 10, 11). Analysis with IgG-specific conjugate revealed that significantly higher level of RB51-specific IgG was present at 6 weeks p.i. in mice vaccinated with RB51 and RB51WbkA when compared with saline-inoculated mice (Figure 10A). Moreover, mice vaccinated with RB51 and RB51WbkA developed significantly higher levels of IgG2a, IgG2b, and IgG3 isotypes specific to RB51 at 3 and 6 weeks p.i. when compared with saline-inoculated controls (Figures 10C–E). However, only vaccination with strain RB51WbkA resulted in a significant increase in RB51-specific IgG1 antibody at 6 weeks p.i. when compared with the saline-inoculated group of mice (Figure 10B).

Significantly increased levels of RB51WbkA-specific IgG, IgG2a, and IgG3 antibodies were detected in serum of mice vaccinated with strains RB51 and RB51WbkA at 3 and 6 weeks p.i. than in saline-inoculated mice (Figures 11A,C,E). Moreover, mice vaccinated with RB51WbkA developed significantly higher levels of RB51WbkA-specific IgG at 3 and 6 weeks p.i., as well as RB51WbkA-specific IgG2a at 6 weeks p.i., when compared with the RB51 vaccinated group of mice (Figures 11A,C). Only vaccination of mice with RB51WbkA resulted in significantly increased levels of RB51WbkA-specific IgG1 at 3 and 6 weeks p.i. when compared with the saline-inoculated mice (Figure 11B). Assay with IgG2b-specific conjugate revealed that RB51WbkA-specific IgG2b antibody was present at significantly higher levels in RB51 and RB51WbkA vaccinated groups of mice at 6 weeks p.i. when compared to saline-inoculated controls. However, at 3 weeks p.i., only the mice vaccinated with strain RB51WbkA developed significantly higher levels of RB51WbkA-specific IgG2b antibody than the saline-inoculated group (Figure 11D).

### Protection against challenge with virulent B *abortus* 2308

**Figure 12:**
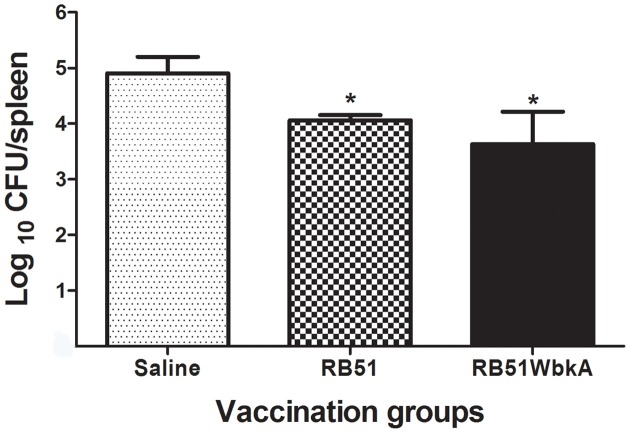
**Detection of the protective efficacy of RB51WbkA against challenge with the virulent strain** ***B. abortus*** **2308**. Mice vaccinated with RB51, recombinant RB51WbkA and saline-inoculated mice were challenged by i.p. inoculation of 3 × 10^4^ CFU-equivalent of virulent strain 2308. Two weeks post-challenge, the mice were euthanized and the *Brucella* CFUs in their spleens were determined. Results are shown as mean ± standard deviation (*n* = 5) of the log CFU of *Brucella* recovered from spleens. ^*^Significantly different from the corresponding saline group (*P* < 0.01).

Mice vaccinated with RB51 and RB51WbkA had significantly reduced number of virulent *B. abortus* 2308 in their spleens when compared with the saline-inoculated group of mice (Figure 12). However, there was no statistical difference in the splenic bacterial loads between the two vaccinated groups of mice.

## Discussion

In this study, we unexpectedly discovered that overexpression of *wbkA* in strain RB51 confers it an extreme mucoid and clumping phenotype that is associated with the production of a yet to be characterized sugar-based polymer with many properties of canonical EPS. The structure of EPS produced by RB51WbkA still needs to be determined. However, the qualitative results of our lectin binding studies indicate the presence of mannose, galactose, N-acetylglucosamine, and N-acetylgalactosamine in the EPS. This EPS composition is similar to that reported for *B. melitensis* (Godefroid et al., 2010), where mannose was detected to be the primary sugar in the EPS. Several of our attempts to purify the EPS from RB51WbkA, following a previously described procedure (Godefroid et al., 2010), were unsuccessful. We failed to precipitate the EPS. This could have been because of the molecular nature of the complexes between EPS and rough LPS of RB51. Any future studies should take this into consideration for developing strategies for purification of EPS from RB51WbkA.

It is known that *wbkA*, which encodes a putative glycosyltransferase, is essential for the O-PS and smooth LPS synthesis in *B. abortus* and *B. melitensis* (Gonzales et al., 2008). However, the present study is the first to demonstrate the role of this gene product in EPS production in a *Brucella* strain. The molecular basis for this unexpected observation is at present, unknown. To date, production of EPS was reported in certain recombinant gene-knockout or gene-overexpression strains of *B. melitensis* (Uzureau et al., 2007; Godefroid et al., 2010; Wang et al., 2010; Mirabella et al., 2013). *B. melitensis* strain deficient in transcriptional regulator VjbR, which is involved in quorum sensing (QS), was shown to produce EPS-like substance (Uzureau et al., 2007). Overexpression of AiiD, an enzyme that degrades QS molecule acylhomoserine lactone (AHL), also leads to production of EPS in *B. melitensis* (Godefroid et al., 2010). Interestingly, overexpression of MucR, an orthologue of a *Sinorhizobium meliloti* transcriptional regulator of its succinoglycan EPS, in *B. melitensis* resulted in clumping phenotype which is associated with EPS production (Mirabella et al., 2013). A study also showed that EPS production occurs in *virB* mutants of *B. melitensis* (Wang et al., 2010). Interestingly, overexpression of WbkA in strain VTRA1, a *B. abortus wboA* mutant (Winter et al., 1996; McQuiston et al., 1999), also resulted in the development of mucoid colonies that formed strings in liquid culture (result not shown). However, overexpression of WbkA in *B. abortus* 2308 resulted in mucoid colonies on solid media but the bacteria did not form strings in liquid culture (result not shown). This suggests that only overexpression of *wbkA* in *wboA*-deficient genetic background results in alteration of surface properties. Currently, it is not clearly known if *Brucella* produces EPS in response to any natural environmental conditions or during the course of infection in mammalian hosts. However, above mentioned studies document the genetic competence of *B. melitensis* to produce EPS. Evidence for clumping phenotype and EPS production in *B. melitensis* under certain hypertonic culture conditions (Mirabella et al., 2013), and in *B. abortus* under microaerobic conditions (Almiron et al., 2013) was also reported.

*Brucella* belongs to the class *Alphaproteobacteria*; several members of the class *Alphaproteobacteria* are known to produce EPS(s) during their life-cycle (Leigh et al., 1985; Stredansky and Conti, 1999). For example, *S. meliloti* produces an EPS, succinoglycan, which is essential for the expression of its full virulence including formation and invasion of nodules (Leigh et al., 1985; Leigh and Coplin, 1992). Interestingly, a family of glycosyltransferases was shown to be required for the synthesis of succinoglycan by *Sinorhizobium* spp (Glucksmann et al., 1993). Similarly, *Agrobacterium* strains also produce an EPS which is structurally identical to the succinoglycan EPS; however, it has been found to be dispensable for the formation of crown gall tumors by *Agrobacterium tumefaciens* (Leigh and Coplin, 1992). The EPS produced by RB51WbkA increased the bacterial adherence to plastic matrix, but it did not have an effect on the bacteria's susceptibility to polymyxin B killing and persistence in mice. This suggests that the EPS did not prevent the rough LPS in the cell wall of RB51WbkA from external chemicals nor it was able to alter the attenuation characteristic of the bacteria.

Two-component systems have been found to regulate the EPS formation in many plant pathogenic bacteria, including *S. meliloti* (Cheng and Walker, 1998; Skorupska et al., 2006). These systems can sense the specific bacterial requirements during pathogenesis and can subsequently regulate the production of EPS. Global regulatory mechanisms, such as the two-component systems, QS, also fine-tune the synthesis and secretion of EPS, leading to an increase in synthesis during nutritional stress and a decrease in EPS synthesis during growth of cells under nutrient sufficient environment. A previous study has demonstrated the formation of clumps in liquid culture by a *vjbR* mutant strain of *B. melitensis* (Uzureau et al., 2007). Mutation in *vjbR* render the bacteria incapable of responding to the presence of AHL. One explanation of this phenomenon is that the bacteria are unable to regulate the *vjbR*-dependent AHL-mediated repression of genes which are involved in clumping (Uzureau et al., 2007). VjbR was found to regulate the EPS synthesis and/or export, and also the production of several outer membrane proteins (OMPs) (Uzureau et al., 2007). Another study documented the induction of a similar clumping phenotype by *Brucella* overexpressing AHL-acylase *aiiD* (Godefroid et al., 2010). The authors hypothesize that the overexpression of *aiiD* leads to the degradation of all of the intrinsically synthesized AHLs, resulting in unbound VjbR regulators which activate the expression of genes involved in clumping (Godefroid et al., 2010). *wbkA* was found to be a QS-target using proteomic and microarray analysis (Uzureau et al., 2010). It is interesting that WbkA is the only glycosyltransferase identified as a target of QS-regulators in the study. Further studies need to be undertaken to investigate the role of the QS-regulators in modulating the expression of *wbkA* under specific environmental conditions and the effect of gene-interplay on the level of the EPS production.

Mice immunized with strain RB51WbkA appeared to develop antibodies to the EPS, as significantly higher levels of IgG antibodies were detected to be specific to RB51WbkA than RB51. IgG1 and IgG2a were the prominent isotypes that contributed to this difference. Nevertheless, both RB51 and RB51WbkA vaccines induced the same level of protective response against the virulent *B. abortus* challenge, suggesting the minimal, if any, role of EPS in modulating immune responses in mice.

In conclusion, our studies demonstrate that the overexpression of *wbkA* in RB51 results in the production of EPS that confers increased adherence property to the polystyrene surfaces. This EPS was found to contain mannose, galactose, N-acetylglucosamine and N-acetylgalactosamine. This finding adds to the growing evidence for the EPS synthesis in *Brucella*. EPS has previously been shown to aid some bacteria to survive in a hostile environment, evade the immune mechanism of the host, and adhere to the host cells (Zhu et al., 2001; D'Haeze and Holsters, 2004). Further studies to identify the role of EPS in affecting the bacterial fitness under different environmental conditions would help delineate the precise contribution of EPS to the pathogenesis of *Brucella*.

## Author contributions

Conceived and designed the experiments: ND and RV. Performed the experiments: ND, MS, NG, and NS. Analyzed the data: ND, MS, NG, NS, and RV. Contributed to the writing of the manuscript: ND and RV.

### Conflict of interest statement

The authors declare that the research was conducted in the absence of any commercial or financial relationships that could be construed as a potential conflict of interest.

## Abbreviations

CFU: Colony forming unit
OD: Optical density.
